# Impact of Direct Acting Antivirals on Survival in Patients with Chronic Hepatitis C and Hepatocellular Carcinoma

**DOI:** 10.1038/s41598-019-53051-2

**Published:** 2019-11-19

**Authors:** William M. Kamp, Cortlandt M. Sellers, Stacey Stein, Joseph K. Lim, Hyun S. Kim

**Affiliations:** 1 Section of Interventional Radiology, Department of Radiology and Biomedical Imaging, Yale School of Medicine, 330 Cedar Street, New Haven, CT 06510 USA; 2 Section of Medical Oncology, Department of Internal Medicine, Yale School of Medicine, 330 Cedar Street, New Haven, CT 06510 USA; 3 Section of Digestive Diseases and Yale Liver Center, Yale School of Medicine, 330 Cedar Street, New Haven, CT 06510 USA; 4 Yale Cancer Center, Yale School of Medicine, New Haven, CT 06510 USA

**Keywords:** Gastroenterology, Hepatocellular carcinoma

## Abstract

With the increasing use of direct-acting antivirals (DAA) for treatment of chronic hepatitis C virus (HCV) infection, we looked at the impact of DAA use and 12-week sustained viral response (SVR12) in patients with hepatocellular carcinoma (HCC) and HCV. This is a retrospective analysis of 969 HCC patients diagnosed from 2005 to 2016 at an urban tertiary-care hospital. Kaplan-Meier curves and multivariable Cox proportional hazards models were used to assess survival. Median overall survival of the cohort was 24.2 months. 470 patients had HCV (56%). 123 patients received DAA therapies for HCV (26.2%), 83 of whom achieved SVR12 (67.4%). HCV-positive and HCV-negative patients had similar survival, 20.7 months vs 17.4 months (p = 0.22). Patients receiving DAA therapy had an overall survival of 71.8 months vs 11.6 months for patients without (p < 0.0001). DAA patients who achieved SVR12 had an overall survival of 75.6 months vs. 26.7 months in the non SVR12 group (p < 0.0001). Multivariable analysis revealed AJCC, Child-Pugh Score, MELD, tumor size, tumor location, cancer treatment type, receiving DAA treatment and achieving SVR12 had independent influence on survival (p < 0.05). This suggests DAA therapy and achieving SVR12 is associated with increased overall survival in HCV patients with HCC.

## Introduction

Hepatocellular carcinoma (HCC) represents a global public health burden, affecting an estimated 14 million persons worldwide, and is the third leading cause of cancer mortality^1^. Within the United States, HCC is ranked 7^th^ for cancer related mortality and has seen a doubling in incidence from 1975 to 2007^1–4^. The primary predisposing factors for HCC carcinogenesis is liver cirrhosis^1^. Cirrhosis risk factors include chronic alcohol use, viral hepatitis, including hepatitis c (HCV), and non-alcoholic fatty liver disease^1^.

Chronic HCV infection is the second most common risk factor for HCC and is responsible for 10–25% of all HCC cases^1^. Over 20–30 years, 20–30% of patients with chronic HCV infections will develop cirrhosis and end stage liver disease and 1–4% of these patients will progress to HCC each year^5,6^. Of all HCV related HCC cases, 80–90% occur in the setting of cirrhosis^2^. With more than 3.5 million HCC patients in the United States and an estimated 130–170 million patients worldwide currently infected with HCV, the importance of HCV management in HCC therapeutic care and prevention is clear^7,8^. The major current therapeutic goal for HCV and prevention of liver disease progression is sustained viral response (SVR), which is defined by negative HCV RNA at 12 weeks post-treatment (SVR12) and appears to be durable with a late virologic relapse rate of less than 1%^9,10^.

Therapeutic management of HCV has recently shifted from interferon-based therapies to all-oral interferon-free direct-acting antiviral (DAA) combination regimens. DAAs are a new class of drugs that target nonstructural proteins responsible for replication and infection of the hepatitis c virus^10–12^. Genotype specific DAA therapies have been shown to reach SVR12 exceeding 90% of patients with fewer adverse effects compared with historic interferon-based regiments^7,13–18^. SVR12 from DAA regimens have been associated with a decrease in liver outcomes including cirrhosis, hepatic decompensation, HCC and mortality^19^. However, the impact of DAA regimens on clinical outcomes in patients with HCC remain limited. This study evaluates the impact of DAA on overall survival in HCV patients with HCC with the *a priori* hypothesis that SVR12 would be associated with improved outcome.

## Materials and Methods

### Study cohort

The protocols and methods were conducted in compliance with the Health Insurance Portability and Accountability Act and were approved by the Yale institutional review board. The institutional review board approved this study without requiring additional patient consent. Patients diagnosed with radiologic or histopathologic HCC diagnosis defined by NCI/AASLD guidelines from 2005 to 2016 were retrospectively identified from the institutional cancer registry of a single urban academic center. Treatment allocation had been determined by a multi-disciplinary tumor board. Patients were grouped by primary HCC treatment including liver transplantation, tumor resection, interventional oncological procedures (catheter-based therapies, ablation or combination locoregional therapy), systemic management (chemotherapy or radiation) and supportive care (palliative or no treatment). All patients were reviewed for history of HCV infection diagnosis based on positive HCV antibody, positive HCV RNA and/or ICD-9 recorded in electronic medical records. Only those with a HCV infection diagnosis were assessed for DAA treatment. Coinfections such as hepatitis B (HBV) and/or human immunodeficiency virus (HIV) or prior HCV treatment with therapies other than DAA and/or use of multiple DAAs did not preclude patients from analysis. SVR12 status was only collected for patients with HCV plus any reported DAA use for HCV. Patients that reached SVR12 on interferon-based regimens were not included with patients reaching SVR12 via DAA regimens. Exclusion criteria for this study included age less than 18 years, unknown survival status, and histopathologic diagnosis of combined HCC and cholangiocarcinoma. Liver transplant patients were excluded from HCV, DAA and SVR12 overall survival and multivariable hazard ratio analyses. Patients were conservatively excluded in analyses for which they had unknown values. The primary outcome of interest was overall survival (OS), defined as time from HCC diagnosis to all-cause mortality or censoring.

### Statistical analysis

Kaplan-Meier curves and Cox proportional hazards models were used to assess survival. Univariate analysis of age, sex, Child-Pugh Score, tumor size, model for end-stage liver disease (MELD), AJCC stage, body mass index, alpha-fetoprotein level, platelet count, unilobar or bilobar tumor presentation, presence of multiple tumors, main treatment, HCV infection, DAA treatment and SVR12 status were performed and variables with a p-value < 0.1 were included into multivariable analysis. Statistical analyses performed with JMP Pro 13.1.0 (SAS Institute, Cary, North Carolina) and GraphPad Prism 8.0.0 (GraphPad Software, La Jolla, California). Values were considered statistically significant with a p-value of less than 0.05.

## Results

### Cohort description

**Figure 1:**
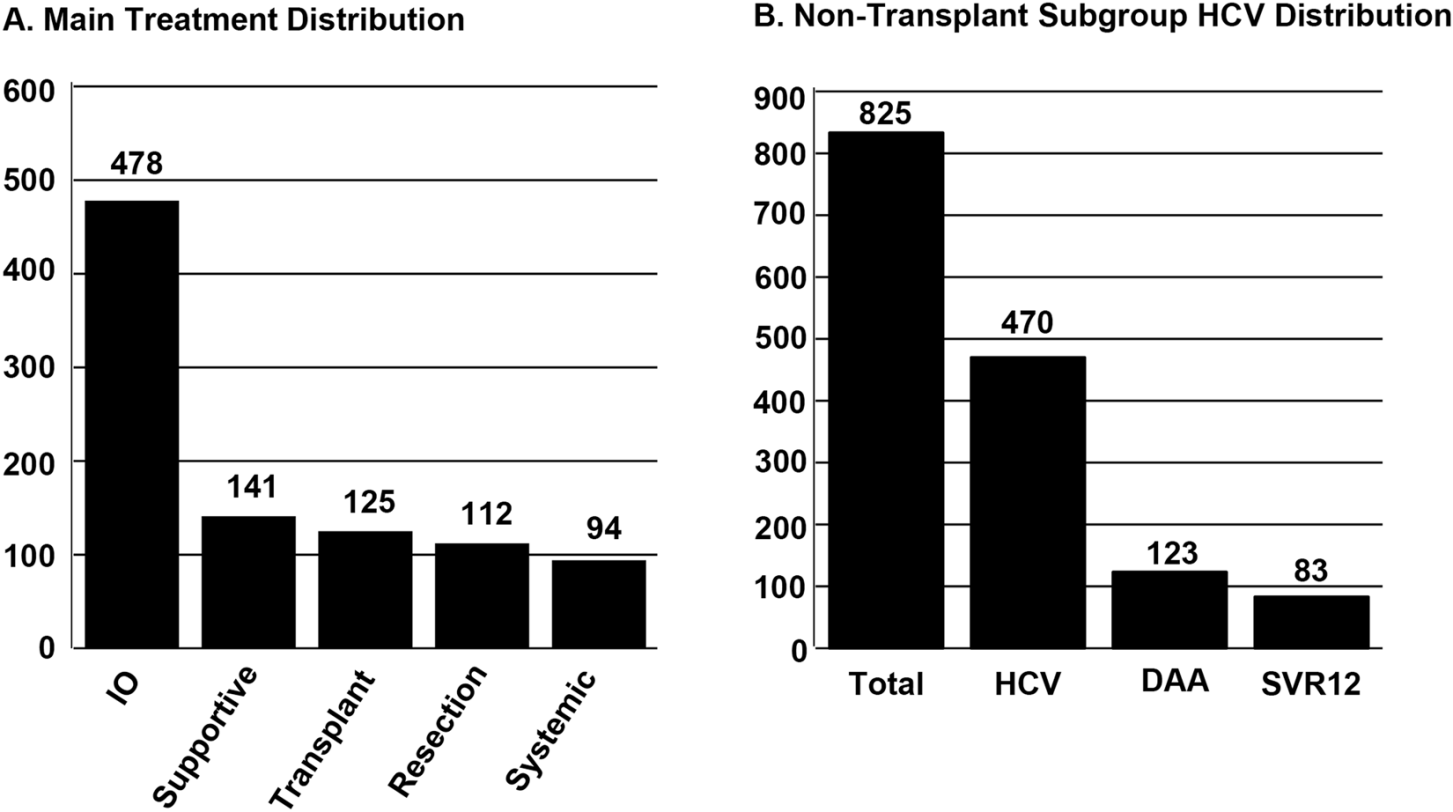
(**A**) Treatment allocation of the entire hepatocellular carcinoma cohort. (**B**) Patients stratified by HCV infection, DAA therapy, and achievement of SVR12.

**Figure 2:**
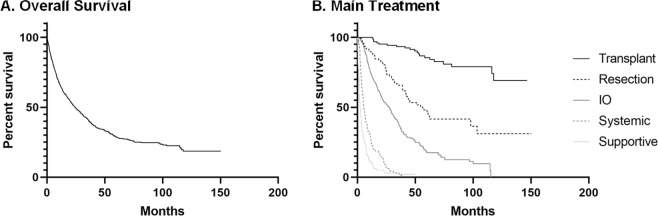
(**A**) Survival rate for all hepatocellular carcinoma (HCC) patients within cohort (n = 969) in months since HCC diagnosis. (**B**) OS for HCC patients by main HCC treatment method. Patients receiving liver transplantation (n = 125). Patients undergoing tumor resection (n = 112), interventional oncology (IO) (n = 478), systemic therapy (n = 94) and supportive management (n = 141) (overall p < 0.0001).

Between 2005 and 2016, 969 HCC patients met inclusion criteria (Table 1). Mean age of cohort at HCC diagnosis was 62.8 ± 10.2 years. The group was predominately male at 79%. Median time of follow-up from HCC diagnosis to death or final encounter in electronic health record was 17.4 months (interquartile range (IQR): 6.7–38.0) for the cohort, 14.4 months (IQR: 6.5–31.0) for HCV patients, 25.7 months (IQR: 13.2–38.3) for patients receiving DAA and 27.7 months (IQR: 13.8–42.3) for patients reaching SVR12. As shown in Fig. 1A, 478 patients (49.3%) received interventional oncology therapies, 141 (14.6%) received supportive care, 125 (12.9%) underwent liver transplantation, 112 (11.6%) had tumor resection and 94 (9.7%) received chemotherapy and/or radiation as their primary treatment. Among non-transplant patients, 470 (57.0%) patients were HCV positive. Twenty-seven patients had HIV coinfection, 19 had hepatitis B coinfection and 4 patients had both HIV and hepatitis B coinfections. Only 123 (26.2%) of the HCV positive patients received a DAA treatment regimen. DAAs were administered prior to HCC diagnosis in 25.7% of HCV patients and post-HCC diagnosis in 74.3% patients. 61.3% of patients that received DAAs after their HCC diagnosis began DAA therapy within one year of the HCC diagnosis. Mulitple DAA regimens were used within the cohort including ledipasvir/sofosbuvir (53.8%), sofosbuvir alone (15.4%), sofosbuvir/simeprevir (8.5%), telaprevir (6%), sofosbuvir/velpatasvir (2.6%), and six other regimens used in only one patient each (5.1%). Of those patients receiving DAAs for HCV treatment, 83 (67.4%) achieved SVR12 (Fig. 1B). Median OS for all patients was 24.2 months (95% CI:20.9–27.9) (Fig. 2A). Median OS for patients receiving liver transplantation (n = 125) was not reached as more than 50% of subgroup were alive at time of last follow up. Patients undergoing tumor resection (n = 112) had a median OS of 56.7 months (95% CI: 41.9–103.5), interventional oncology (IO) (n = 478) median OS of 27.7 months (95% CI:22.3–30.7), systemic therapy (n = 94) median OS of 5.6 months (95% CI: 4.4–7.3) and supportive management (n = 141) median OS of 2.4 months (95% CI: 1.9–3.2, overall p < 0.0001, Fig. 2B).

**Table 1:** Cohort and Subgroup Characteristics.

	Cohort	HCV Patients	Non-DAA Patients	DAA Patients	Non-SVR12 Patients	SVR12 Patients
Age at HCC Diagnosis (years) (mean ± stdev)	62.8 ± 10.2	60.5 ± 7.7	59.9 ± 7.7	61.7 ± 5.8	61.6 ± 5.3	61.8 ± 6.1
Sex						
Male	768 (79.3%)	390 (83.0%)	202 (82.4%)	99 (80.5%)	28 (90.3%)	63 (75.9%)
Female	201 (20.7%)	80 (17.0%)	43 (17.6%)	24 (19.5%)	3 (9.7%)	20 (24.1%)
**Liver Factors**						
AJCC						
1	400 (43.8%)	198 (44.5%)	79 (34.5%)	79 (65.3%)	15 (48.4%)	58 (71.6%)
2	244 (26.7%)	110 (24.7%)	63 (27.5%)	31 (25.6%)	11 (35.5%)	19 (23.5%)
3	162 (17.7%)	86 (19.3%)	53 (23.1%)	8 (6.6%)	3 (9.7%)	4 (4.9%)
4	107 (11.7%)	51 (11.5%)	34 (14.8%)	3 (2.5%)	2 (6.5%)	0
Child Pugh Score						
A	512 (55.2%)	254 (56.3%)	116 (49.2%)	84 (68.9%)	24 (77.4%)	54 (65.9%)
B	284 (30.6%)	132 (29.3%)	80 (33.9%)	30 (24.6%)	6 (19.4%)	21 (25.6%)
C	132 (14.2%)	65 (14.4%)	40 (16.9%)	8 (6.6%)	1 (3.2%)	7 (8.5%)
MELD (mean ± stdev)	11.4 ± 5.7	10.7 ± 4.6	11.4 ± 5.2	9.5 ± 3.4	9.3 ± 3.1	9.67 ± 3.5
Tumor Size (cm) (mean ± stdev)	4.48 ± 3.6	4.18 ± 3.3	4.70 ± 3.6	2.76 ± 1.7	3.09 ± 2.4	2.57 ± 1.3
Tumor Location						
Unilobar	628 (66.7%)	298 (65.1%)	137 (57.6%)	93 (75.6%)	17 (54.8%)	68 (81.9%)
Bilobar	314 (33.3%)	160 (34.9%)	101 (42.4%)	30 (24.4%)	14 (45.2%)	15 (18.1%)
Multiple Tumors						
yes	442 (45.9%)	225 (48.4%)	133 (55.0%)	47 (38.2%)	19 (61.3%)	27 (32.5%)
no	520 (54.1%)	240 (51.6%)	109 (45.0%)	76 (61.8%)	12 (38.7%)	56 (67.5%)
**Main Treatment**						
Transplant	125 (13.2%)	0	0	0	0	0
Resection	112 (11.8%)	56 (12.1%)	21 (8.6%)	21 (17.4%)	5 (16.1%)	16 (19.5%)
IO	478 (50.3%)	285 (61.6%)	143 (58.4%)	93 (76.9%)	24 (77.4%)	63 (76.8%)
Systemic	94 (9.9%)	45 (9.7%)	26 (10.6%)	4 (3.3%)	1 (3.2%)	1 (1.2%)
Supportive	141 (14.8%)	77 (16.6%)	55 (22.4%)	3 (2.5%)	1 (3.2%)	2 (2.4%)
**Hepatitis C Factors**						
HCV Infection	551 (56.9%)	470	245	123	31	83
DAA Treatment		123 (26.2%)	0	123	31	83
SVR12				83 (67.5%)	0	83

Characteristics of cohort, HCV, non-DAA, DAA, non-SVR12 and SVR12 subgroups, n (%) or mean ± standard deviation as marked. AJCC: American Joint Committee on Cancer stage, MELD: model for end stage liver disease, IO: interventional oncology, HCV: hepatitis C, DAA: direct-acting antivirals, SVR12: sustained viral response at 12 weeks.

### Overall survival in HCV and DAA subgroups

**Figure 3:**
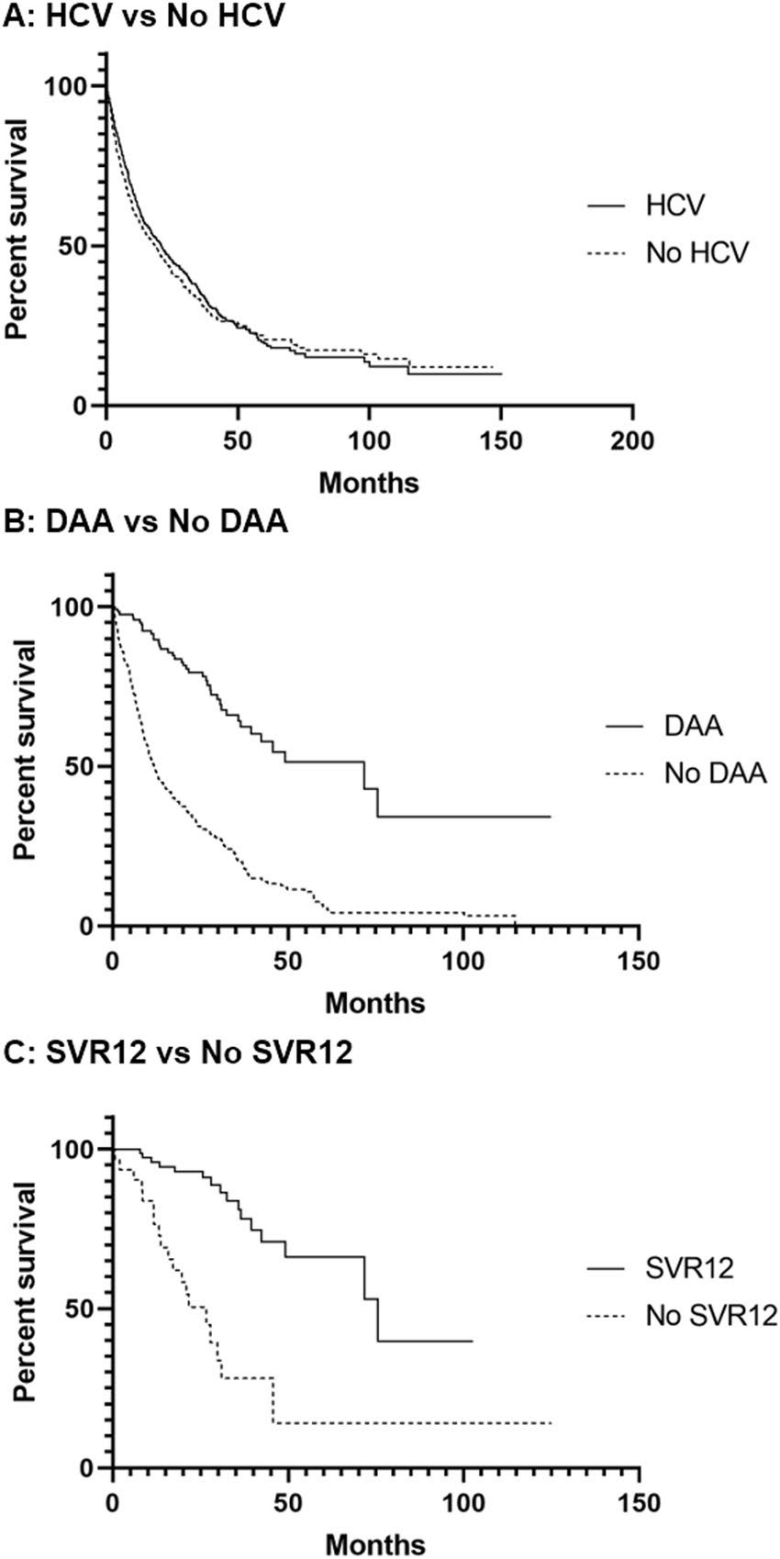
Patient survival rate in months since hepatocellular carcinoma (HCC) diagnosis. (**A**) HCC Patients with positive history of hepatitis c (HCV) infection (n = 470) versus patients with no history of HCV (n = 363) (p = 0.22). (**B**) HCC and HCV patients that received a direct-acting antiviral (DAA) (n = 123) versus those who did not receive a DAA (n = 247) (p < 0.0001). (**C**) Patients with HCC and HCV that received a DAA and achieved sustained viral response (SVR12) (n = 83) versus those who did not achieve SVR12 (n = 31) (p < 0.0001).

Subgroup analysis of HCV patients, recipients of DAA and those that achieved SVR12 revealed significant influences on OS. Although patients with and without HCV showed no significant difference in survival: median OS 20.7 months (95% CI: 16.5–24.1) versus 17.4 months (95% CI: 13.0–20.6) respectively, (p = 0.22) (Fig. 3A), HCV patients that received DAA had a median OS of 71.8 months (95% CI: 39.5-not reached) compared to 11.6 months (95% CI: 9.8–14.5) for HCV patients that did not use DAAs (p < 0.0001) (Fig. 3B). Patients achieving SVR12 had a higher median OS of 75.6 months (95% CI: 49.2-not reached) versus 26.7 months (95% CI: 13.7–31.1) for patients with positive HCV RNA by PCR 12 weeks post-DAA cessation (Fig. 3C) (p < 0.0001).

### Prognostic factors

**Figure 4:**
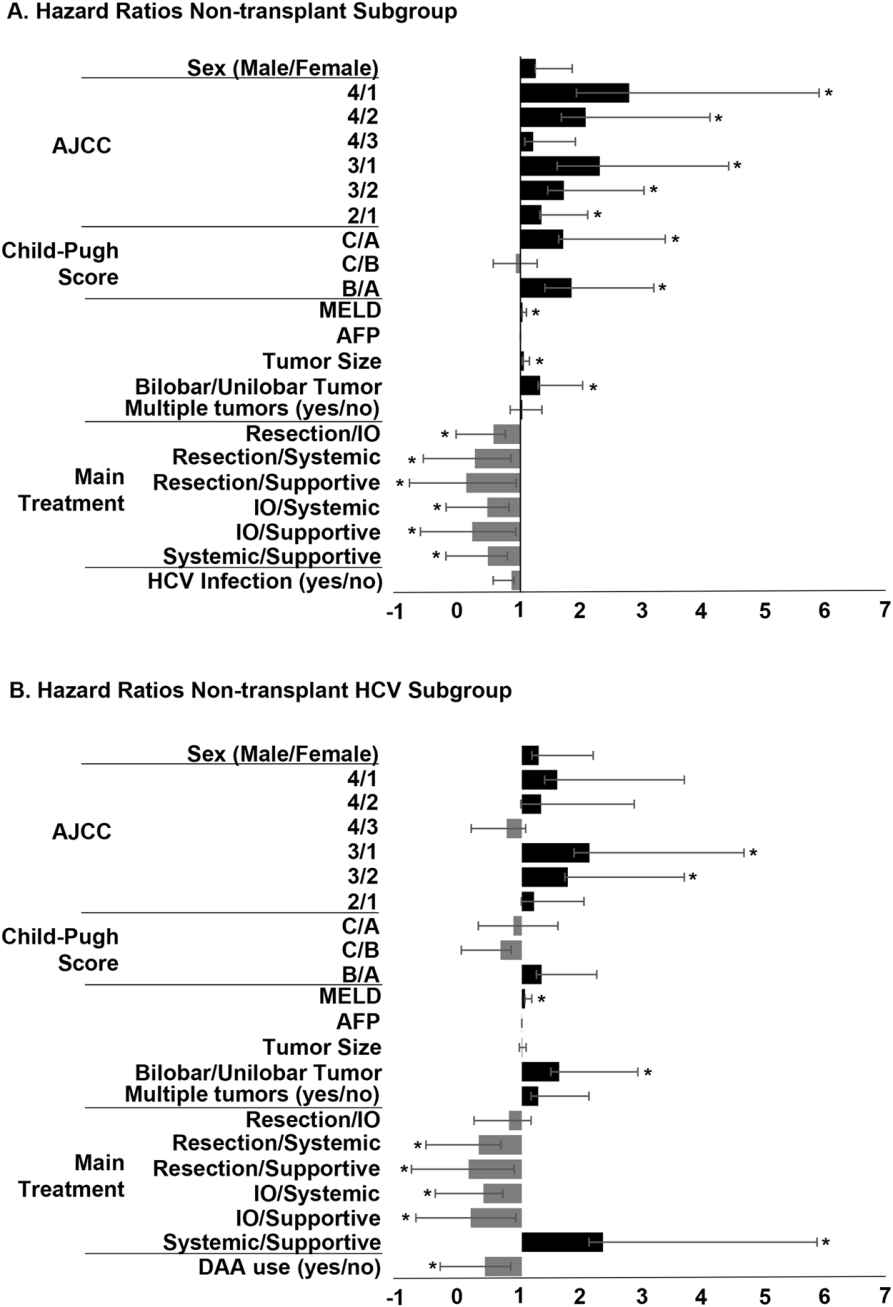
Hazard ratios from multivariable analysis on overall survival in non-transplant hepatocellular carcinoma (HCC) patients. Values greater than one indicate increased risk of death. Values less than one indicate reduced risk of death. (**A**) Hazard ratios in all non-transplant HCC patients. (**B**) Hazard ratios in all non-transplant HCC with history of HCV. *p < 0.05, AJCC: American Joint Committee on Cancer stage, MELD: model for end stage liver disease, AFP: alpha-fetoprotein, IO: interventional oncology, HCV: hepatitis C, DAA: direct-acting antivirals.

Multivariable analyses of subgroups were performed to assess impact of relevant HCC prognostic markers, HCC therapies, HCV factors and lab values. Within all non-transplant patients, numerous factors significantly influenced overall survival (Fig. 4A, Table 2). AJCC stage 4 had decreased survival compared to stage 1 (HR = 2.77, 95% CI:1.86–4.08, p < 0.0001) and 2 (HR = 2.06, 95% CI: 1.40–3.01, p = 0.0003). AJCC stage 3 also had poorer survival as compared to stage 1 (HR = 2.29, 95% CI: 1.26–2.30, p < 0.0001) and 2 (HR = 1.70, 95% CI: 1.26–2.30, p = 0.0005). AJCC stage 2 had a HR = 1.34 (95% CI: 1.03–1.75, p = 0.03) compared to stage 1. Increased Child-Pugh score was also associated with poorer survival (Child Pugh C vs. A: HR 1.69, 95% CI: 1.07–2.65, p = 0.02; B vs A: HR 1.83, 95% CI: 1.43–2.233, p < 0.0001), as were increased MELD score, increased tumor size, and bilobar tumors (p < 0.05). Treatment allocation significantly impacted survival, with resection demonstrating improved survival vs interventional oncology (HR = 0.56, 95% CI: 0.39–0.81, p = 0.002), systemic therapies (HR = 0.26, 95% CI:0.16–0.42, p < 0.0001) and supportive management (HR = 0.12, 95% CI:0.08–0.19, p < 0.0001). Interventional oncology treatment increased survival rates over systemic therapies (HR = 0.46, 95% CI:0.33–0.65, p < 0.0001) and supportive management (HR = 0.22, 95% CI:0.16–0.29, p < 0.0001). Systemic therapies showed improved survival rates over supportive therapies (HR = 0.47, 95% CI:0.42–0.68, p < 0.0001).

**Table 2:** Hazard ratios for cohort, HCV subgroup and DAA subgroup.

	Total	HCV	DAA
Sex - Male/Female	1.25 (0.99–1.49, p = 0.0592)	1.28 (0.89–1.89, p = 0.1924)	1.30 (0.39–5.25, p = 0.6799
**Liver Factors**			
MELD	**1**.**03 (1**.**00–1**.**06**, **p** = **0**.**0168)**	**1**.**05 (1**.**00–1**.**11**, **p** = **0**.**0468)**	1.21 (1.02–1.44, p = 0.0326)
Tumor Size	**1**.**06 (1**.**02–1**.**09**, **p** < **0**.**0007)**	1.01 (0.95–1.06, p = 0.08082)	0.75 (0.49–1.06, p = 0.1044)
Bilobar/Unilobar	**1**.**32 (1**.**03–1**.**69**, **p** = **0**.**026)**	**1**.**62 (1**.**14–2**.**30**, **p** = **0**.**0072)**	1.38 (0.36–5.51, p = 0.6354)
Multiple Tumors - yes/no	1.03 (0.80–1.32, p = 0.816)	1.27 (0.88–1.83, p = 0.1986)	1.01 (0.41–2.99, p = 0.9831)
AJCC			
4/1	**2**.**77 (1**.**86–4**.**08**, **p** < **0**.**0001)**	1.59 (0.79–3.09, p = 0.19)	
4/2	**2**.**06 (1**.**40–3**.**01**, **p** = **0**.**0003)**	1.32 (0.67–2.53, p = 0.4209)	
4/3	1.21 (0.86–1.68, p = 0.2704)	0.75 (0.42–1.31, p = 0.3186)	
3/1	**2**.**29 (1**.**69–3**.**09**, **p** < **0**.**0001)**	**2**.**11 (1**.**25–5**.**54**, **p** < **0**.**0051)**	1.81 (0.08–13.48, p = 0.6350)
3/2	**1**.**70 (1**.**26–2**.**30**, **p** = **0**.**0005)**	**1**.**76 (1**.**05–2**.**92**, **p** = **0**.**0328)**	2.28 (0.10–19.85, p = 0.5336)
2/1	**1**.**34 (1**.**03–1**.**75**, **p** = **0**.**0305)**	1.20 (0.79–1.82, p = 0.3872)	0.80 (0.27–2.22, p = 0.6658)
Child-Pugh Score			
C/A	**1**.**69 (1**.**07–2**.**65**, **p** = **0**.**0235)**	0.86 (0.42–1.73, p = 0.6775)	0.13 (0.01–1.55, p = 0.1068)
C/B	0.93 (0.63–1.35, p = 0.6904)	0.65 (0.36–1.18, p = 0.1549)	0.15 (0.01–1.67, p = 0.1234)
B/A	**1**.**83 (1**.**43–2**.**33**, **p** < **0**.**0001)**	1.33 (0.92–1.91, p = 0.1351)	0.85 (0.30–2.40, p = 0.7584)
**Main Treatment**			
Resection/IO	**0**.**56 (0**.**39–0**.**81**, **p** = **0**.**0019)**	0.79 (0.42–1.36, p = 0.4118)	0.39 (0.05–1.92, p = 0.2649)
Resection/Systemic	**0**.**26 (0**.**16–0**.**42**, **p** < **0**.**0001)**	**0**.**29 (0**.**13–0**.**64**, **p** = **0**.**002)**	
Resection/Supportive	**0**.**12 (0**.**08–0**.**19**, **p** < **0**.**0001)**	**0**.**12 (0**.**06–0**.**25**, **p** < **0**.**0001)**	0.12 (0.00–5.53, p = 0.2586)
IO/Systemic	**0**.**46 (0**.**33–0**.**65**, **p** < **0**.**0001)**	**0**.**37 (0**.**21–0**.**68**, **p** = **0**.**0017)**	
IO/Supportive	**0**.**22 (0**.**16–0**.**29**, **p** < **0**.**0001)**	**0**.**16 (0**.**10–0**.**26**, **p** < **0**.**0001)**	0.31 (0.01–9.82, p = 0.4652)
Systemic/Supportive	**0**.**47 (0**.**32–0**.**68**, **p** < **0**.**0001)**	**2**.**34 (1**.**23–4**.**52**, **p** = **0**.**0093)**	
**Hepatits C Factors**			
HCV Infection - yes/no	0.85 (0.70–1.04, p = 0.1151)		
DAA Therapy - yes/no		**0**.**39 (0**.**26–0**.**58**, **p** < **0**.**0001)**	
SVR12 Attained - yes/no			**0**.**14 (0**.**06–0**.**35**, **p** < **0**.**0001)**

Hazard ratios from multivariable analysis. HR, 95% Confidence Interval, p-value, AJCC: American Joint Committee on Cancer stage, MELD: model for end stage liver disease, IO: interventional oncology, HCV: hepatitis C, DAA: direct-acting antivirals, SVR12: sustained viral response at 12 weeks.

Multivariable analysis of non-transplant HCV patients showed similar results to non-transplant patients (Fig. 4B) with the addition of DAA use as a significant prognostic marker (HR = 0.39, 95% CI:0.26–0.58, p < 0.0001). In the final subgroup, including only DAA patients, only two factors were significant, MELD (HR = 1.21, 95% CI:1.02–1.44, p = 0.03) and SVR12 (HR = 0.14, 95% CI:0.06–0.35, p < 0.0001).

## Discussion

As incidence and prevalence of HCC increases, understanding the impact of HCV treatment with DAA on long term outcomes in this population is vital. Approximately one-half of cases among the three-fold increase in HCC incidence between 1975 and 2007 in the US can be attributed to the aging chronic HCV population^2^. Although there are indications that DAAs may slow progression to HCC^20,21^, there remains a vast population of HCC patients that could potentially benefit from treatment of their chronic HCV infections.

It is likely that the improved median overall survival seen in our cohort among HCC patients receiving DAA treatment is a direct result of the high success rate of achieving SVR12. Although more patients are needed to reduce the possible influence of DAA exclusion from patients with worse prognoses, the over three-fold difference in median overall survival between those that did and did not achieve SVR12 likely indicates profound longitudinal effects of HCV cure in HCC patients. Although our data indicates less severe HCC and liver disease in DAA and SVR12 patients versus their subgroup counterparts, multivariable analysis supports reduced all-cause mortality in patients receiving DAAs and achieving SVR12. In addition, others have reported that achieving SVR12 is associated with improved liver function, Child-Pugh scores and reversal of liver decompensation symptoms which could also be factors in the improved survival^22,23^. Recently other investigators have shown similar results to those presented in this study. They report increased overall survival in patients with complete response to HCC therapy treated with DAA^24,25^. Singal *et al*. reported that at 31 health centers in US and Canada the risk of death was substantially lower in patients receiving DAA with effects hinging on those achieving SVR^24^.

Reaching SVR12 is not an easy task in the HCC patient population. Only 69% of our population reached SVR12 with similar results in other retrospective analyses of DAA use in HCC patients^26,27^ compared to reported values of over 90% in populations powered to DAA efficacy^18^. Part of this may be due to the difficulty of integrating HCV treatment into HCC care, as demonstrated by the low rate of HCC patients with HCV receiving DAA treatment in our cohort (<50%). While there is currently much discussion as to how aggressive clinicians should be about treating active HCV in HCC patients, no official guidelines currently exist^23,28^. Frequently, HCC management supersedes HCV treatment as many providers seek to triage HCV therapy until after the cancer has been treated^23^. In addition, debate is ongoing as to whether or not DAA use increases HCC recurrence rates^18,20,28–31^.

Interestingly, our results suggest that there is ample time for HCV intervention in newly diagnosed HCC patients. The DAA treatment course typically lasts 8–24 weeks^32,33^, and patients receiving resection or IO treatments, 74% of our cohort population, had a median overall survival greater than 27 months. We are hopeful that conversion from non-DAA to DAA medications and subsequent SVR12 achievement will increase as awareness and use of DAA in current HCV management increases.

Our findings are subject to several limitations. These results are based in a retrospective analysis and are therefore prone to sample selection biases; in particular, patients receiving treatment for HCV had less severe liver disease. In addition, our data was limited to chart review of our institution’s electronic medical records system. This limited patient sample size, patient follow up and details of patient characteristics at HCC diagnosis of those enrolled in our older medical records systems. This analysis also did not account for changing practices and improved effectiveness of cancer therapies and prognostic tools over the enrollment period. While the magnitude of the effect of DAAs are possibly magnified in our study, they remain in line with results in other recent investigations^24,25^.

This cohort also raises important questions for future research. The patients achieving SVR12 with DAA include those that had previously failed interferon-based therapies and subsequently received DAA after their introduction, those that received multiple courses and combinations of DAA and those that received DAA for different lengths of time. Some received DAA during HCC management while others received DAA prior to their HCC diagnosis. In addition, understanding the efficacy of DAA therapy in HCV patients with HIV and/or HBV coinfections is of particular interest as the mechanism for carcinogenesis and liver disease is unique with each virus^34,35^. More work is needed in areas concerning the differences in DAA regimen efficacy to understand the most appropriate combination of DAA and regimen length and then to tailor these by HCV genotype, HCC prognosis and other host and genetic factors in order to help more patients achieve SVR12 and better overall outcomes.
